# The Rich and Good-Doing

**Published:** 1873-07

**Authors:** 


### THE RICH AND GOOD-DOING.

JAY COOKE, the banker, has a large Bible class of laboring men, whom he meets on Sundays in a church near Philadelphia, and gives the lessons as much time and thought for preparation as if he were employed in great monied transactions.

He has also a beautiful country seat on an island in one of the lakes, to which he invites clergymen to spend a fortnight as his guests, a dozen or more at a time; and when they leave others come, who have not the means of paying for excursions from home; and no doubt he feels thankful that the means and the disposition have been given him to act as the steward of the Master, in thus ministering to the health, and comfort, and enjoyment of those ambassadors from the court of Heaven; and doubtless, too, he considers it, as he ought to do, an honor and a privilege to be permitted to enjoy the association, companionship and conversation of these good men, while their affectionate and sincere prayers for him will be a thousand fold compensation to him for his thoughtful consideration of those whose high calling it is to preach the Gospel.

A Roman Catholic gentleman, whose name does not occur at present, has done the same thing for the priests of his Church, and it is to be hoped that their good example will be followed by others of other denominations all over the country. To all persons who have country seats we say, take some worthy clergyman along with you for two or three weeks, and when the time is out have another, and be sure their presence will carry blessings to your household, as the Ark of the Covenant carried blessings to the man who took charge of it, "to him and to all his."

How comes it that the wealthy gentlemen above named are thus thoughtful of the ministers of the Gospel? Doubtless it was from the teachings of pious mothers and the influences of the services of the sanctuary on the Sabbath day. Very likely those sainted mothers are in heaven to-day, and little dreamed, when they were nursing their little ones, and caring for them all through childhood, that they were raising up money kings, who should become nursing fathers to the Church and its ministers. Let every young mother who reads this have the lessons constantly before her own mind, and let it be an encouragement to all to labor and to wait patiently in faith, that the "end cometh" when they "shall have their reward," in that their life-work was not "wood, hay, stubble," but gold, and silver, and precious stones. But suppose you "care for none of these things." Your sons, if they attain wealth, will use it at the club house, in ostentatious Sunday drives to the Central Park, in snobbish exhibitions of "four in hand" at summer watering places, in criminal dalliances in the green room of the theatre and in fast living, generally to wind up with a premature death by "heart disease," a result of high living and expensive wines, after having begotten children whose minds and bodies have been impregnated with moral and physical diseases, to be perpetuated in coming generations. Surely the measure of parental responsibility borders on the infinite.

